# Experimental colitis in mice is attenuated by topical administration of chlorogenic acid

**DOI:** 10.1007/s00210-015-1110-9

**Published:** 2015-03-07

**Authors:** Hubert Zatorski, Maciej Sałaga, Marta Zielińska, Aleksandra Piechota-Polańczyk, Katarzyna Owczarek, Radzisław Kordek, Urszula Lewandowska, Chunqiu Chen, Jakub Fichna

**Affiliations:** 1 Department of Biochemistry, Faculty of Medicine, Medical University of Lodz, Mazowiecka 6/8, 92-215 Lodz, Poland; 2 Department of Gastroenterological Surgery, Tenth People’s Hospital of Shanghai, School of Medicine, Tongji University, Shanghai, China; 3 Department of Pathology, Faculty of Medicine, Medical University of Lodz, Lodz, Poland

**Keywords:** Chlorogenic acid, Trinitrobenzenesulfonic acid, Experimental colitis, Inflammatory bowel diseases, Crohn’s disease, Ulcerative colitis

## Abstract

Epidemiological data suggest that the consumption of polyphenol-rich foods reduces the incidence of cancer, coronary heart disease, and inflammation. Chlorogenic acid (CGA), an ester of caffeic and quinic acids, is one of the most abundant polyphenol compounds in human diet with proven biological effectiveness both in vitro and in vivo. The aim of the study is to investigate the possible anti-inflammatory effect of CGA in the gastrointestinal (GI) tract and its mechanism of action. We used a well-established model of colitis, induced by intracolonic (i.c.) administration of trinitrobenzenesulfonic acid (TNBS) in mice. The anti-inflammatory effect of CGA in the colon was evaluated based on the clinical and macroscopic and microscopic parameters. To investigate the mechanism of protective action of CGA, myeloperoxidase (MPO), H_2_O_2_, and NF-κB levels were assessed in the colon tissue. CGA administered i.c. at the dose of 20 mg/kg (two times daily) protected against TNBS-induced colitis more effectively than the same dose administered orally (p.o.), as evidenced by significantly lower macroscopic and ulcer scores. Furthermore, CGA (20 mg/kg, i.c.) reduced neutrophil infiltration, as demonstrated by decreased MPO activity. Moreover, CGA suppressed activation of NF-κB, as evidenced by lower levels of phospho-NF-κB/NF-κB ratio in the tissue. CGA did not affect the oxidative stress pathways. CGA exhibits anti-inflammatory properties through reduction of neutrophil infiltration and inhibition of NF-κB-dependent pathways. Our results suggest that CGA may have the potential to become a valuable supplement in the treatment of GI diseases.

## Introduction

Inflammatory bowel diseases (IBDs), which include Crohn’s disease (CD) and ulcerative colitis (UC), are chronic, relapsing inflammatory disorders of the gastrointestinal (GI) tract. Whereas the inflammation in UC affects only colonic mucosa, in CD, it affects the entire gut wall and may occur in any part of the GI tract (Podolsky 2002). Currently, the incidence and prevalence of IBD are the highest in westernized nations (Molodecky et al. 2012). However, considering that the incidence of IBD is increasing, mortality is low, and the disease is most often diagnosed in the young people, the prevalence of IBD will continue to increase globally (Molodecky et al. 2012). Treatment with 5-aminosalicylates, immunosuppressive agents, glucocorticosteroids, and biological therapies are generally effective in IBD. However, the side effects and economic costs, especially with biological therapy, cannot be ignored. These facts, together with impaired patients’ quality of life, imply that new therapeutics are needed.

The etiology of IBD remains largely unknown, but it is considered to be related to a combination of genetic, microbial, immunological, and environmental factors that results in an inordinate and abnormal immune response in genetically vulnerable individuals. Recent studies highlight that both innate and adaptive immune responses maintain the same importance in development of gut inflammation (Zhang and Li 2014). However, apart from the presence of activated innate immune response cells, such as macrophages, neutrophils, and monocytes, an excessive production of reactive oxygen species (ROS) and pro-inflammatory mediators, including TNF-α and interleukin (IL)-1, -6, and -8, plays a major role in the pathogenesis of IBD (Babior 2000; Sun et al. 2013). Of note, natural immunity, and also most types of inflammation, is mediated by transcription nuclear factor κB (NF-κB)-related pathways (Umezawa 2011). It has been shown that NF-κB is strongly activated in patients with IBD, as well as in experimental models of colitis (Atreya et al. 2008).

**Fig. 1:**
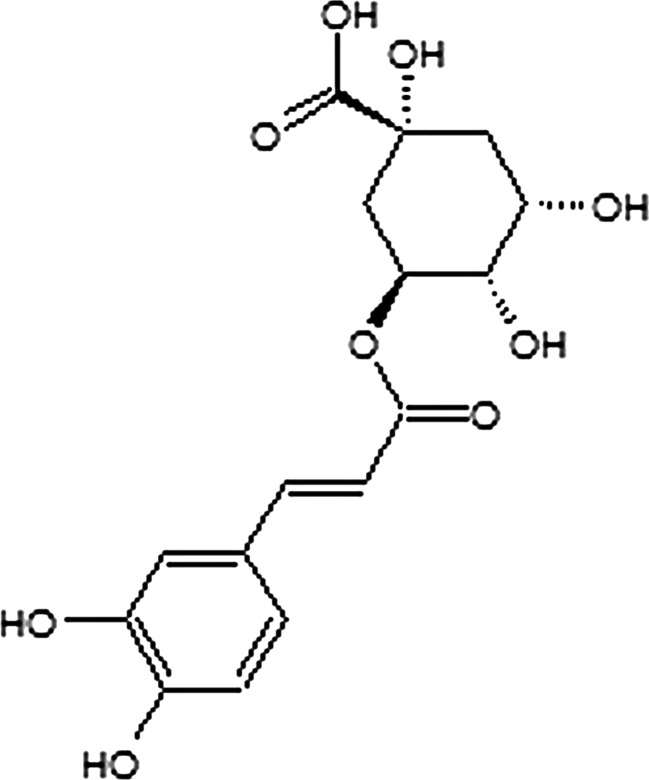
Chemical structure of CGA

Chlorogenic acid (CGA), formed by esterification of caffeic and quinic acids (Fig. 1), is one of the most abundant polyphenols in nature (Suzuki et al. 2006). CGA widely occurs in medicinal plants from Europe and Asia, for example, honeysuckle, *Angelicae sinensis Radix*, and *Chuanxiong Rhizoma*, as well as in coffee beans and apples (Hebeda et al. 2011; Hulme 1953; Li et al. 2014a, b). Several studies were conducted to investigate biological activities of CGA, including its anti-bacterial, anti-carcinogenic, and anti-oxidant effects in animal models of diseases (Kim et al. 2010; Lafay et al. 2006; Ruifeng et al. 2014); however, the effect of CGA on the inflammatory reaction in the GI tract has not been explored so far.

In search for novel therapeutics and supplements for treatment of GI diseases, here we characterized the anti-inflammatory action of CGA in experimental colitis in mice. In particular, we were interested in the effect of CGA after disparate routes of administration, as well as the involvement of NF-κB-regulated pathways. The animal model of colitis used in our experiments, induced by intracolonic (i.c.) administration of 2,4,6-trinitrobenzenesulfonic acid (TNBS), has been chosen for this study because it closely mimics clinical and morphological features of human CD (Elson et al. 1995).

## Materials and methods

### Animals

Male balbC mice were obtained from the Animal House at the University of Lodz, Poland. All animals, weighing 22–28 g, were housed at a constant temperature (22 °C) and maintained under a 12-h light/dark cycle in sawdust-lined plastic cages with free access to chow and tap water. All animal protocols were approved by the Medical University of Lodz Animal Care Committee (Protocol #670/2012).

### Induction of colitis

Colitis was induced by i.c. administration of TNBS, as described previously (Storr et al. 2009). Briefly, mice were lightly anesthetized with 1 % isoflurane (Baxter Healthcare Corp., IL, USA) and TNBS (4 mg in 0.1 mL of 30 % ethanol in saline) was administered into the colon through a catheter inserted 3 cm proximally to the anus. Previous experiments showed that the dose of TNBS used in this study induced reproducible colitis.

### Pharmacological treatment

CGA was administered two times daily at the dose of 20 mg/kg orally (p.o., 150 μL) and i.c. (100 μL), with the first treatment 30 min before the induction of colitis in acute colitis model. In semi-chronic colitis model, CGA was administered two times daily at the dose of 20 mg/kg i.c. (100 μL) from day 3 to day 6. CGA was dissolved in 5 % dimethyl sulfoxide (DMSO) in saline, which was used as vehicle. Control animals received vehicle alone. Briefly, mice were lightly anesthetized with 1 % isoflurane (Baxter Healthcare Corp., IL, USA) and CGA was administered into colon through a catheter inserted 3 cm proximally to the anus.

### Evaluation of colonic damage

Animals were killed by cervical dislocation 3 days after TNBS application in acute colitis model and 7 days post-TNBS in semi chronic colitis model. Colons were instantly removed, opened longitudinally, washed with phosphate-buffered saline (PBS), and immediately examined. Macroscopic colonic damage was assessed using an established semi-quantitative scoring system by adding individual scores for ulcer, colonic shortening, wall thickness, and presence of hemorrhage, fecal blood, and diarrhea, as described previously (Fichna et al. 2012; Sałaga et al. 2013, 2014b). For ulcer score and colonic shortening, the following scale was used: ulcer, 0.5 points for each 0.5 cm; shortening of the colon, 1 point for >15 % and 2 points for >25 % (based on the mean length of the colon in untreated mice of 8.07 ± 0.20 cm, *n* = 6). The wall thickness was measured in millimeters with vernier caliper, and thickness of *n* in mm corresponds to *n* scoring points. The presence of hemorrhage, fecal blood, or diarrhea increased the score by 1 point for each additional feature. The macroscopic scoring was performed in blind manner.

### Histology

After macroscopic scoring, segments of the distal colon were stapled flat, mucosal side up, onto cardboard and fixed in 10 % neutral-buffered formalin for 24 h at 4 °C. Samples were then dehydrated, embedded in paraffin, sectioned at 5 μm, and mounted onto slides. Subsequently, sections were stained with hematoxylin and eosin and examined using a Motic AE31 microscope (Ted Pella, Redding, CA, USA). Photographs were taken using a digital imaging system consisting of a digital camera (Moticam 2300, Ted Pella, Redding, CA, USA) and image analysis software (MoticImages Plus 2.0, Motic Deutschland GmbH, Wetzlar Germany). Microscopic total damage score was determined in blind manner based on the presence (score = 1) or absence (score = 0) of goblet cell depletion, the presence (score = 1) or absence (score = 0) of crypt abscesses, the destruction of mucosal architecture (normal = 1, moderate = 2, extensive = 3), the extent of muscle thickening (normal = 1, moderate = 2, extensive = 3), and the presence and degree of cellular infiltration (normal = 1, moderate = 2, transmural = 3).

### Determination of tissue myeloperoxidase activity

The method adapted by Fichna et al. (2012) was used to assess granulocyte infiltration and to quantify the myeloperoxidase (MPO) activity. Shortly, 1-cm segments of colon were weighed and homogenized in hexadecytrimethylammonium bromide (HTAB) buffer (0.5 % HTAB in 50 mM potassium phosphate buffer, pH 6.0; 50 mg tissue/mL) immediately after isolation. Homogenate was centrifuged (15 min, 13,200 rpm, 4 °C), and 7 μL of supernatant was added to each well on a 96-well plate, containing 200 μL of 50 mM potassium phosphate buffer (pH 6.0), supplemented with 0.167 mg/mL of O-dianisidine hydrochloride and 0.05 μL of 1 % H_2_O_2_. Absorbance was measured at 450 nm (iMARK Microplate Reader, Biorad, Hemel Hempstead, Hertfordshire, UK). All measurements were performed in triplicate. MPO was expressed in milliunits per gram of wet tissue, 1 unit being the quantity of enzyme able to convert 1 μmol of H_2_O_2_ to water in 1 min at room temperature. Units of MPO activity per 1 min were calculated from a standard curve using purified peroxidase enzyme.

### Determination of tissue hydrogen peroxide levels

Assay was performed according to the methodology described before by Sałaga et al. (2014a).

Briefly, 50 mg of colon tissue fragments was homogenized with 2 mL of 1.15 % potassium chloride. Then, 10-μL aliquots of tissue homogenate were mixed with 90 μL of PBS (pH 7.0) and 100 μL of horseradish peroxidase (1 U/mL) containing 400 μmol homovanilic acid (HRP+HVA assay) or with 90 μL of PBS and 100 μL of 1 U/mL horseradish peroxidase only (HRP assay). Both homogenates were incubated for 60 min at 37 °C. Subsequently, 300 μL of PBS and 125 μL of 0.1 M glycine-NaOH buffer (pH 12.0) with 25 mM EDTA were added to each homogenate sample. Excitation was set at 312 nm and emission was measured at 420 nm (PerkinElmer Luminescence Spectrometer, Beaconsfield, UK). Readings were converted into H_2_O_2_ concentration using the regression equation *Y* = 0.012*X* − 0.007, where *Y* = H_2_O_2_ concentration in homogenate (μM) and *X* = intensity of light emission at 420 nm for HRP+HVA assay reduced by HRP assay emission (arbitrary units, AU). The regression equation was prepared from three series of calibration experiments with 10 increasing H_2_O_2_ concentrations (range 10–1000 μM). The lowest H_2_O_2_ detection was 0.1 nM, with intra-assay variability not exceeding a 2 %.

### Western blot analysis of NF-κB protein levels

Shortly, the tissue was minced and homogenized with the use of a motor-driven Potter homogenizer in 10 volumes of lysis buffer. Concentration of total protein pool was evaluated in each sample using modified Lowry protocol (Cadman et al. 1979). Separation of proteins (30 μg/well) was performed on 7.5 % SDS-PAGE gel in electrophoretic buffer. Electrophoretically separated proteins were electroblotted using Immobilon P membranes (pore size, 0.45 μm) in transfer buffer. The membranes were incubated for 1 h at room temperature in 3 % non-fat dry milk Tris-buffered saline with Tween 20 (0.05 %) to saturate non-specific protein binding sites (Stawinska et al. 2008). The membranes were probed with the following primary antibodies (Santa Cruz Biotechnology, Paso Robles, CA, USA) overnight at 4 °C: anti-rabbit anti-mouse p65 (1:4000) and pp65 (1:5000) and β-GAPDH (1:3000) as reference protein. Appropriate horseradish-peroxidase-conjugated secondary antibodies were applied for 1 h at room temperature, and then the bands were visualized using a 3,3′,5,5′-12 tetramethylbenzidine (TMB) as a substrate for the localization of HRP activity. Qualitative and quantitative analysis was performed by measuring integrated optical density (OD) by GelProAnalyzer ver. 3.0 for Windows^TM^ program (Media Cybernetics, Warrendale,, PA, USA).

### Drugs

All drugs and reagents, unless otherwise stated, were purchased from Sigma-Aldrich (Poznan, Poland). CGA (purity 98 % by high-performance liquid chromatography) was extracted from honeysuckle flowers and purchased from Nanjing Zelang Medical Technology Co. (Nanjing, Jiangsu, China) by one of the authors (C.C.). In all in vivo tests, drugs were dissolved in 5 % DMSO in saline, which was used as vehicle in control groups. Vehicle affected none of the measured parameters when given alone.

### Statistics

Statistical analysis was performed using Prism 5.0 (GraphPad Software Inc., La Jolla, CA, USA). The data are expressed as means ± SEM. One-way ANOVA followed by Newman-Keuls post hoc test was used for analysis. *P* values <0.05 were considered statistically significant.

## Results

### Intracolonic administration of CGA attenuates acute TNBS-induced colitis in mice

**Fig. 2:**
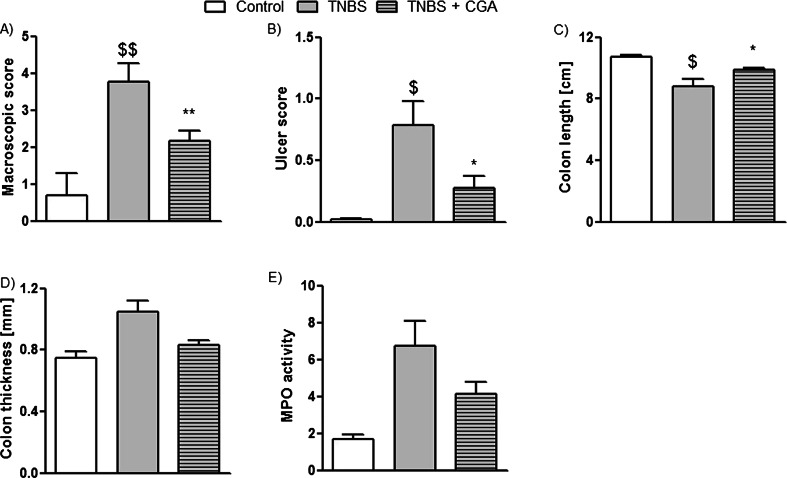
CGA (20 mg/kg) injected two times daily i.c. over 3 days attenuated TNBS-induced colitis in mice. Shown are data for macroscopic scores (**a**), ulcers score (**b**), colon length (**c**), colon thickness (**d**), and MPO activity (**e**) after i.c. administration. ^$^ *P* < 0.05, ^$$^ *P* < 0.01, as compared to control (vehicle treated) mice; *P < 0.05, **P < 0.01, as compared to TNBS-treated animals. Data represent mean ± SEM of 7–12 mice per group

**Fig. 3:**
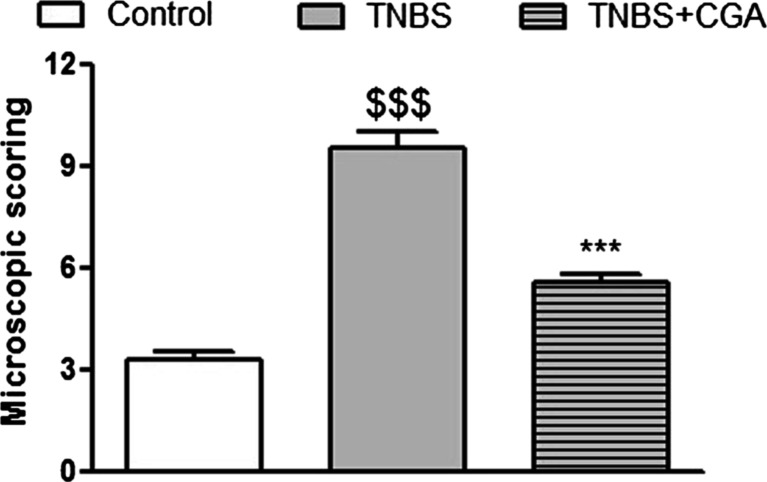
Microscopic total damage score were attenuated by injection of CGA (20 mg/kg, two times daily, i.c.). ^$$$^ *P* < 0.001, as compared to control (vehicle-treated) mice; ***P < 0.001 as compared to TNBS-treated animals. Data represent mean ± SEM of 7–12 mice per group

The i.c. instillation of TNBS caused reproducible colitis in mice, characterized by elevated macroscopic colon damage score and increased MPO activity. The i.c. administration of CGA (20 mg/kg, two times daily) significantly attenuated acute TNBS-induced colitis, as demonstrated by decreased total macroscopic score (2.16 ± 0.27 vs. 3.77 ± 0.50 for CGA+TNBS- and TNBS-treated mice, respectively) (Fig. 2a). Moreover, CGA significantly reduced ulcer score (0.29 ± 0.09 vs. 0.79 ± 0.19 for CGA+TNBS- and TNBS-treated mice, respectively) (Fig. 2b) and increased mouse colon length compared with TNBS-treated animals (Fig. 2c). Colon thickness and MPO activity were reduced by i.c. administration of CGA, but the differences did not reach statistical significance (Fig. 2d, e, respectively). Histological evaluation of colon damage revealed that i.c. CGA significantly reduced the microscopic score (Fig. 3). Administration of CGA in dose of 20 mg/kg i.c. had no effect on studied parameters in control mice.

### CGA administered p.o. has anti-inflammatory effect on acute TNBS-induced colitis in mice

**Fig. 4:**
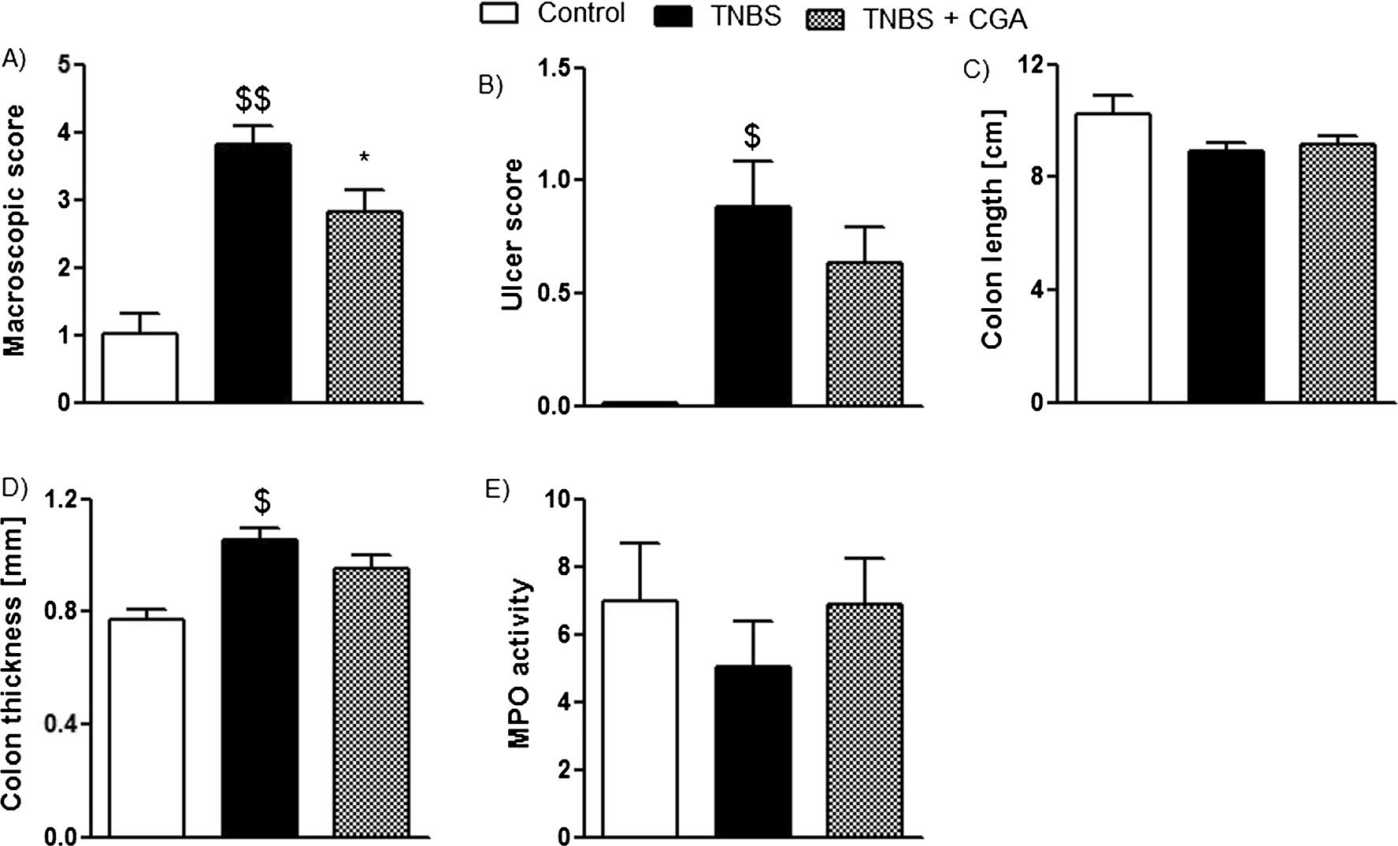
CGA (20 mg/kg) injected two times daily p.o. over 3 days attenuated TNBS-induced colitis in mice. Shown are data for macroscopic score (**a**), ulcer score (**b**), colon length (**c**), colon thickness (**d**), and MPO activity (**e**). ^$^ *P* < 0.05, ^$$^ *P* < 0.01, as compared to control (vehicle-treated) mice; *P < 0.05, as compared to TNBS-treated animals. Data represent mean ± SEM of 7–12 mice per group

**Fig. 5:**
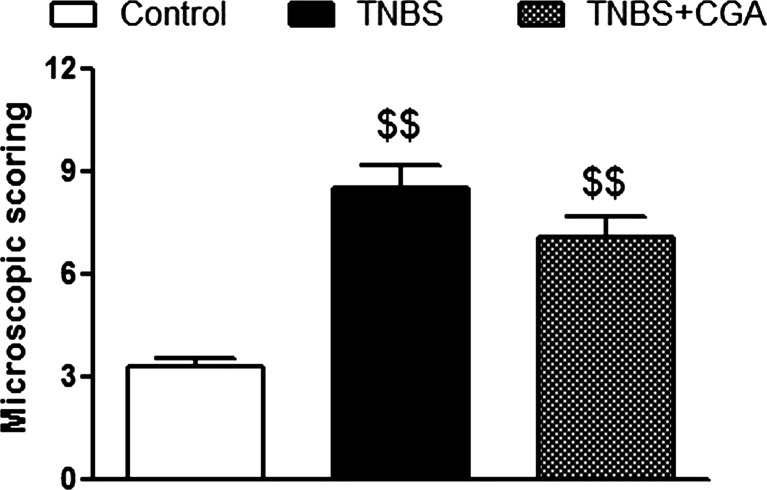
CGA (20 mg/kg) injected two times daily p.o. over 3 days attenuated TNBS-induced colitis in mice. Shown are data from microscopic total damage score. ^$$^ *P* < 0.01, as compared to control (vehicle-treated) mice. Data represent mean ± SEM of 7–12 mice per group

Oral administration of CGA attenuated acute TNBS-induced colitis in mice, but the effect was weaker than that observed after i.c. instillation (macroscopic score, 2.82 ± 0.3 vs. 2.16 ± 0.27 for p.o. and i.c. CGA, respectively, Figs. 4a and 2a; ulcer score, 0.63 ± 0.15 vs. 0.28 ± 0.09 for p.o. and i.c. CGA, respectively, Figs. 4b and 2b). Furthermore, CGA (20 mg/kg, p.o., two times daily) did not have any significant effect on colon length (Fig. 4c), colon thickness (Fig. 4d), and MPO activity (Fig. 4e) in TNBS-treated mice. In addition, p.o. administration of CGA did not attenuate microscopic score (Fig. 5). Administration of CGA in dose of 20 mg/kg p.o. had no effect on studied parameters in control mice.

### Intracolonic administration of CGA attenuates established TNBS-induced colitis in mice

**Fig. 6:**
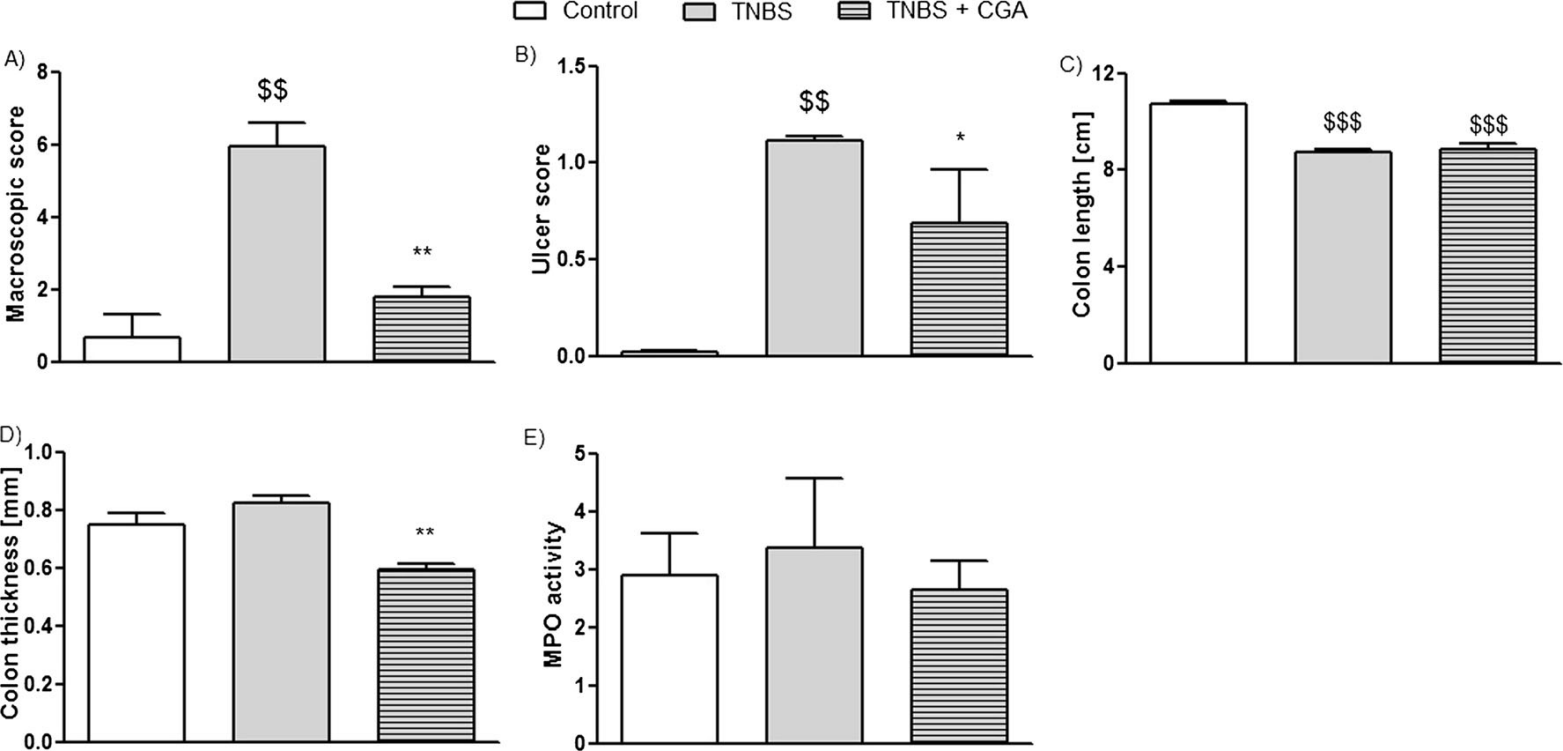
CGA (20 mg/kg) injected two times daily i.c. over 5 days attenuated established TNBS-induced colitis in mice. Shown are data for macroscopic score (**a**), ulcer score (**b**), colon length (**c**), colon thickness (**d**), and MPO activity (**e**). ^$$^ *P* < 0.01, ^$$$^ *P* < 0.001 as compared to control (vehicle-treated) mice; *P < 0.05, **P < 0.01 as compared to TNBS-treated animals. Data represent mean ± SEM of 7–12 mice per group

The i.c. administration of CGA (20 mg/kg, two times daily) significantly attenuated established colonic inflammation in the model of TNBS-induced colitis, as demonstrated by decreased total macroscopic score (1.78 ± 0.28 vs. 5.94 ± 0.67 for CGA+TNBS- and TNBS-treated mice, respectively) (Fig. 6a). Moreover, CGA significantly reduced ulcer score (0.69 ± 0.27 vs. 1.12 ± 0.02 for CGA+TNBS- and TNBS-treated mice, respectively) (Fig. 6b) and decreased mouse colon thickness compared with TNBS-treated animals (Fig. 6d). However, CGA had no effect on colon length (Fig. 6c). Furthermore, MPO activity was slightly reduced, but the difference did not reach statistical significance (Fig. 6e).

### CGA administered i.c. did not affect hydrogen peroxide level in acute TNBS-induced colitis

**Fig. 7:**
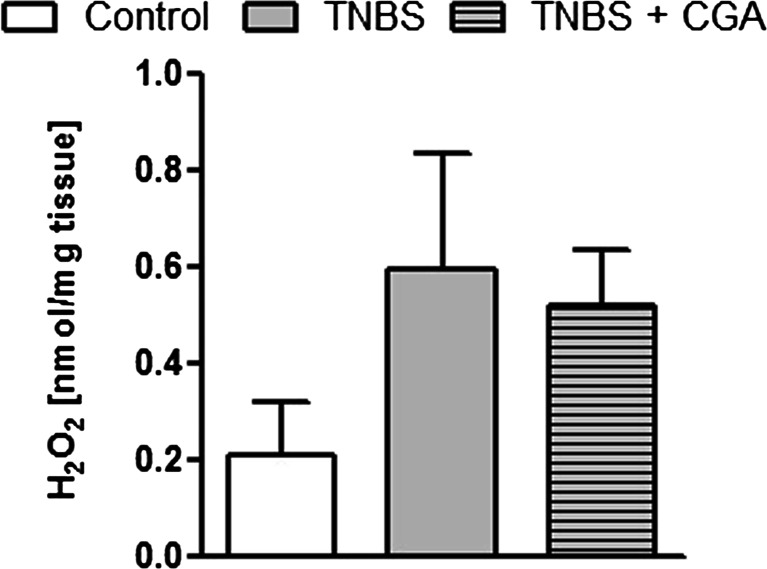
The effect of CGA (20 mg/kg, i.c., two times daily) on hydrogen peroxide levels in TNBS-treated mice. Data represent mean ± SEM of 7–12 mice per group

As shown in Fig. 7, there was an increase in H_2_O_2_ level measured in the colon of TNBS-treated mice versus control (vehicle treated) animals. The i.c. administration of CGA had no effect on tissue H_2_O_2_ level in TNBS-induced colitis (0.51 ± 0.11 vs. 0.63 ± 0.28 for CGA+TNBS- and TNBS-treated mice, respectively).

### CGA administered i.c. reduced activated NF-κB levels

**Fig. 8:**
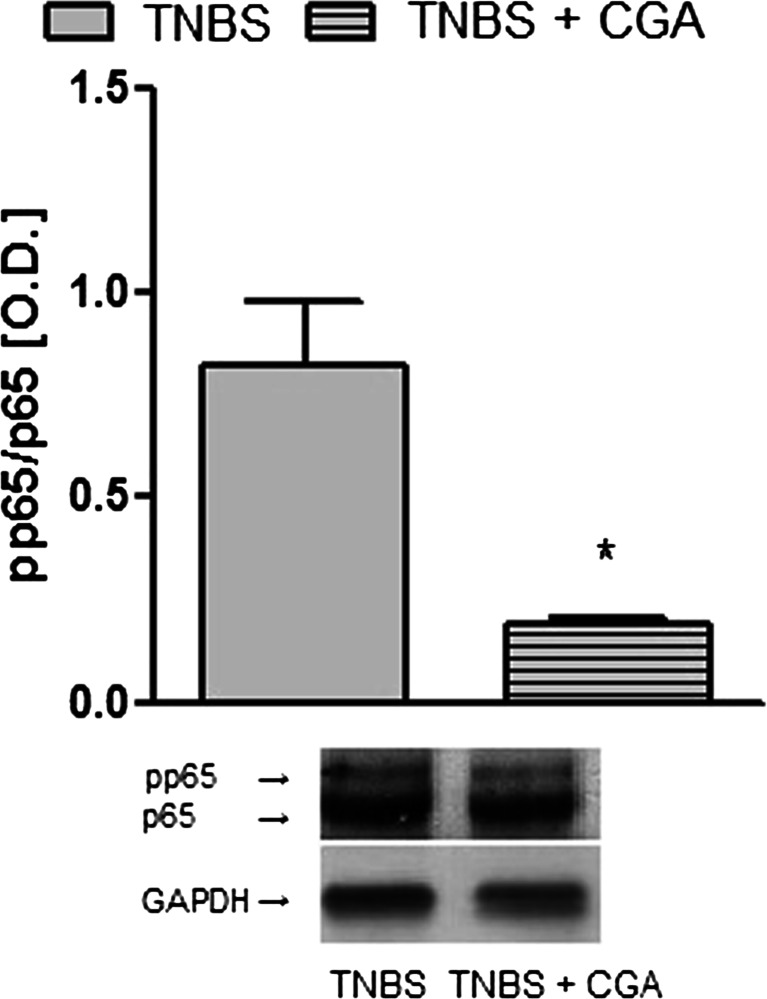
CGA (20 mg/kg, i.c., two times daily) significantly decreased phospho-p65/p-65 ratio in the mouse colon tissue. The *upper panel* shows results of quantitative analysis and the *lower panel* shows a representative blot for protein expression of phosphop65 and p65 in TNBS- and TNBS+CGA-treated mice. *P < 0.05, as compared to TNBS-treated animals. Data represent mean ± SEM of 6–8 mice per group

The i.c. administration of CGA significantly decreased phospho-p65/p-65 ratio in the colon tissue (0.19 ± 0.01 vs. 0.82 ± 0.16 for CGA+TNBS- and TNBS-treated mice, respectively) (Fig. 8).

## Discussion

Comprehensive data in the literature show that polyphenols possess anti-oxidant, anti-carcinogenic, and anti-inflammatory properties, of which only the first two were sufficiently explored. CGA, which is one of the most abundant natural polyphenols, has been proven as a potent anti-oxidant and anti-cancerogenic agent, both in the in vivo and in vitro studies, but only a few reports focused on its anti-inflammatory activity in vivo. Hence, the primary goal of this study was to evaluate the anti-inflammatory effect of CGA and to investigate the potential mechanism of its action in the GI tract.

We showed that CGA displayed a significant anti-inflammatory activity in a well-established mouse model of experimental colitis, as evidenced by reduction of macroscopic damage score, MPO activity, and inhibition of NF-κB activation. We also demonstrated that CGA produced anti-inflammatory effect regardless of the oxidative-stress-related pathways. Our study, along with previously published reports, shows that CGA may become a new therapeutic or an attractive supplement in the treatment of IBD. Furthermore, we showed that the i.c. administration of CGA not only alleviates colitis in the acute phase but also has healing effects on established TNBS-induced colonic inflammation. This indicates that CGA may have both prophylactic and therapeutic activity and may be used in patients regardless of the current stage of the disease. Moreover, the literature data support our observations showing that also other quinic acid derivatives alleviate chronic colitis (di Paola et al. 2010).

Of note, in a study performed by Ng et al. (2014), consumption of coffee had a protective effect against UC development in Asians (adjusted OR 0.51; 95 % CI 0.36 to 0.72; group of 256 UC patients diagnosed between 2011 and 2013 from eight countries in Asia and Australia vs. 940 controls). Furthermore, the average intake of coffee by Americans is 3.1 cups a day; moreover, a single serving of coffee provides between 20 and 675 mg of CGA depending on type of roast and the volume consumed (Del Rio et al. 2010). Thus, the dose of CGA used in this study corresponds to the average intake consumed by human while drinking coffee.

Noteworthy, we found that CGA is more effective after i.c. than p.o. administration in the mouse model of colitis, which speaks for its topical, rather than systemic, application. CGA is absorbed in its intact form in the stomach and—as products of hydrolysis, such as caffeic acid—in the small intestine. Once in the cecum, CGA is fully hydrolyzed into caffeic and other aromatic acids (Lafay et al. 2006), with questionable biologic activity. This in turn results in lower concentration of CGA in inflamed colonic tissue after p.o.—than i.c.—administration, which may be the reason for its weaker anti-inflammatory action.

Immunocyte recruitment and activation are key steps in the intestinal innate immune response (Korzenik et al. 2005; Kucharzik et al. 2001; Reaves et al. 2001), and studies with animal models of colitis highlight the relationship between immunocyte infiltration and disease severity (Knutson et al. 2013). Here we observed that CGA impaired neutrophil recruitment and protected colonic tissue against injury, as evidenced by decreased MPO activity, in line with earlier studies showing a profound effect of CGA on immune cells. For example, Ruifeng et al. (2014) demonstrated that CGA ameliorates damage of mammary tissues by reducing neutrophil infiltration. It was also shown that CGA inhibits pro-inflammatory cytokine release induced by lipopolysaccharide (LPS) in RAW264.7 murine macrophage-like cells (Shan et al. 2009), as well as leukocyte influx and levels of IL-4, IL-5, and TNF-α in serum and bronchoalveolar lavage fluid in endotoxin and allergic lung inflammation (Zhang et al. 2010). A critical point in neutrophil migration is their ability to adhere to endothelial surface and transfer to inflamed tissues by means of over-expressed or activated adhesion molecules. A key role in this process is played by L-selectin located on neutrophils, along with P- and E-selectin on endothelium. A recent study conducted by Hebeda et al. (2011) indicates that CGA treatment blocks L-selectin shedding induced by LPS in neutrophils obtained from rat peritoneum. Moreover, in that study, the treatment with CGA inhibited LPS-induced platelet endothelial cell adhesion molecule 1 (PECAM-1) expression on neutrophil membranes. Our data suggest that the anti-inflammatory effect of CGA in the GI tract may also result from interaction with post-transcriptional mechanisms of adhesion molecule expression (Hebeda et al. 2011).

NF-κB is a key transcription factor involved in promoting the formation of pro-inflammatory mediators. It is a heterodimer consisting of Rel-family proteins including p65, cRel, RelB, p50, and p52 (Hoesel and Schmid 2013). Although NF-κB is indispensable for maintaining immune functions, its excessive activation leads to stimulation of immune cells, resulting in inflammation (Li and Verma 2002). Activation of NF-κB involves phosphorylation of an inhibitory protein IκB, which binds to NF-κB, followed by liberation of activated factor, its entry to the nucleus, and binding to the κB site in DNA (Umezawa 2011). In this study, we investigated the NF-κB activity by assessing pp65/p65 ratio from colonic tissue homogenates. We found that TNBS caused a significant activation of NF-κB, as shown by higher pp65/p65 ratio, which was decreased after CGA treatment. Our observation is in line with the study by di Paola et al. (2010), in which a quinic acid derivative, 3,5-dicaffeoyl-4-maloquinic acid, administered orally reduced MPO levels and inhibited NF-κB activity in experimental colitis induced by i.c. instillation of dinitrobenzenesulphonic acid (DNBS) in rats. In another, in vitro study, the extract from *Cymbopogon citrates*, containing CGA reduced TNF-α levels and NF-κB activity in human macrophages in a monocyte culture (Francisco et al. 2013). Taken together, our study is in line with a growing body of evidence showing that CGA has impact on various types of immune cells via NF-κB-dependent pathways. CGA may thus be used to restore the function of the imbalanced immune system, including GI inflammation.

The molecular mechanism of CGA action in alleviating experimental colitis still requires further research. Recently, oxidative stress and imbalanced production of free oxygen species have been implicated in IBD pathogenesis. Oxidative stress may lead to DNA damage, apoptosis, and cancer development (Valko et al. 2001, 2007). There are several anti-oxidative mechanisms in the GI tract, which include enzymes like catalase (CAT), superoxide dismutase (SOD), and glutathione peroxidase (GPx), along with non-enzymatic scavengers, such as glutathione, transient ions (e.g., Fe^2+^), or flavonoids. SOD catalyses the reduction of O_2_^−^ to H_2_O_2_. In normal cell metabolism, H_2_O_2_ is further reduced by GPx in the presence of NADPH, but during inflammation, when concentration of H_2_O_2_ increases, it is reduced by CAT. Recent studies suggest that peripheral immune cells in patients with active CD have higher SOD activity and H_2_O_2_ production (Beltran et al. 2010). It has also been shown that CGA may be responsible for inhibition of H_2_O_2_-induced apoptotic neuronal death (Kim et al. 2012). In addition, Feng et al. (2005) suggested that CGA may have protective properties against environmental carcinogen-induced carcinogenesis via upregulation of cellular anti-oxidant enzymes and suppression of ROS-mediated NF-κB activation. Another study showed that CGA was effective in protecting PC12 cells against methylmercury (MeHg)-induced damage through decrease of GPx activity and GSH levels (Li et al. 2008). In our study, H_2_O_2_ levels were increased in mice with TNBS-induced colitis compared to control. However, topical administration of CGA did not reduce the amount of H_2_O_2_. The possible explanation is that the dose of CGA was insufficient to produce any effect on high levels of free radicals resulting from inflammatory processes in the mouse colon (Shi et al. 2011). We thus hypothesized that CGA may alter other anti-oxidative pathways, such as inhibition of lipid peroxidation, reduction of DNA damage, or increase of nitric oxide scavenging activity, which may need further confirmation.

## Conclusion

CGA has anti-inflammatory properties in the mouse model of colitis through the reduction of neutrophil infiltration and inhibition of NF-κB-dependent pathways, but not the stimulation of the oxidative stress protection mechanism. Our results suggest that CGA has the potential to become a valuable supplement for the therapy of inflammatory GI diseases.
